# Invasive Mechanical Ventilation Is Associated with Worse Right Ventricular Strain in Acute Respiratory Failure Patients

**DOI:** 10.3390/jcdd11080246

**Published:** 2024-08-09

**Authors:** Shuyuan Wang, Zubair Bashir, Edward W. Chen, Vishnu Kadiyala, Charles F. Sherrod, Phinnara Has, Christopher Song, Corey E. Ventetuolo, James Simmons, Philip Haines

**Affiliations:** 1 Department of Cardiology, First Affiliated Hospital of Nanjing Medical University, Nanjing 210029, China; 2 Department of Ultrasound Medicine, Union Hospital, Tongji Medical College, Huazhong University of Science and Technology, Wuhan 430022, China; 3 Department of Cardiology, Alpert Medical School of Brown University, Providence, RI 02903, USA; 4 Department of Internal Medicine, Yale School of Medicine, New Haven, CT 06510, USA; 5 Department of Cardiology, Healthcare Institute for Innovations in Quality, University of Missouri-Kansas City, Kansas City, MO 64110, USA; 6 Saint Luke’s Mid America Heart Institute, Kansas City, MO 64111, USA; 7 Lifespan Biostatistics, Epidemiology and Research Design, Rhode Island Hospital, Providence, RI 02903, USA; 8 Division of Pulmonary, Critical Care, and Sleep Medicine, Alpert Medical School of Brown University, Providence, RI 02903, USA; 9 Department of Health Services, Policy & Practice, Brown University School of Public Health, Providence, RI 02903, USA

**Keywords:** right ventricular global longitudinal strain, right ventricular free wall longitudinal strain, TAPSE, acute respiratory failure, invasive mechanical ventilation

## Abstract

Right ventricular (RV) dysfunction is associated with poor prognosis in acute respiratory failure (ARF). Our study evaluates the efficacy of RV strain in detecting RV dysfunction in ARF patients requiring invasive mechanical ventilation (IMV) compared to tricuspid annular plane systolic excursion (TAPSE). In this retrospective study involving 376 patients diagnosed with ARF and requiring IMV, we extracted clinical and outcome data from patient records. RV global longitudinal strain (RVGLS), free wall longitudinal strain (FWLS), and TAPSE were measured retrospectively using speckle tracking echocardiography (STE) and traditional echocardiography, respectively. We divided the cohort into three groups: TTE during IMV (TTE-IMV, 223 patients), before IMV (TTE-bIMV, 68 patients), and after IMV (TTE-aIMV, 85 patients). Multivariable regression analysis, adjusted for covariates, revealed significantly higher RVGLS and FWLS in the groups not on IMV at the time of TTE compared to the TTE-IMV group. Specifically, the TTE-bIMV group showed higher RVGLS (β = 7.28, 95% CI 5.07, 9.48) and FWLS (β = 5.83, 95% CI 3.36, 8.31), while the TTE-aIMV group exhibited higher RVGLS (β = 9.39, 95% CI 6.10, 12.69) and FWLS (β = 7.54, 95% CI 4.83, 10.24). TAPSE did not reveal any significant differences across the groups. Our study suggests an association between IMV and lower RVGLS and FWLS in ARF patients, indicating that IMV itself may contribute to RV dysfunction. RVGLS and FWLS appear to be more sensitive than TAPSE in detecting changes in RV function that were previously subclinical in patients on IMV. Prospective studies with TTE before, during, and after IMV are necessary to assess the primary driver of RV dysfunction and to prognosticate STE-detected RV dysfunction in this population.

## 1. Introduction

Right ventricular (RV) dysfunction is associated with adverse clinical outcomes and poor prognosis in various disease states, and the incidence of RV dysfunction in acute respiratory failure (ARF) ranges from 20 to 25% [1,2,3,4,5]. Different methods are used to evaluate and monitor RV function in the medical intensive care unit (MICU), each with its strengths and weaknesses. While invasive hemodynamic monitoring provides real-time information on intrapulmonary and intra-cardiac pressures, it carries an increased risk of complications and does not assess changes in RV contractility [6,7]. The non-invasive assessment of RV function can be challenging due to the complex geometry and retrosternal location of the RV, often obscured by the sternal shadow [8]. Traditional 2-D echocardiography (TTE) is a non-invasive means to evaluate RV function with minimal risk to patients. However, at least two or more echocardiographic parameters are required for an accurate objective multi-parametric assessment of RV function [9], including a gross qualitative visual assessment of RV contractility, tricuspid annular planner systolic excursion (TAPSE), fractional area change (FAC), Doppler S’, and right ventricular index of myocardial performance (RIMP) [9,10,11]. In addition, cardiac MRI (cMRI), which is a non-invasive gold standard for assessing RV function [12,13], is not a practically feasible method to assess RV function in MICU patients. Currently, TAPSE is the most commonly reported quantitative measure for assessing RV function in critical care settings (CCSs). It is angle- and load-dependent and evaluates RV function longitudinally at a single segment of the lateral annulus only [14], thereby missing other important RV segments including the remaining free wall segments (mid and apical free wall segments) [15]. It has also shown reduced sensitivity in detecting RV dysfunction in patients with chronic heart failure and post-cardiac surgery, compared to RVGLS [16,17]. Baron et al. noted TAPSE’s limited ability to identify RV failure, detecting only 36.5% of the cases among septic shock patients requiring mechanical ventilation [18]. In contrast, RVGLS, measured with speckle tracking echocardiography (STE), presents several advantages over TAPSE. It provides a more objective evaluation of RV function, being less angle- and load-dependent [19], and assessing all the RV myocardial segments. Unlike TAPSE, RVGLS is less influenced by RV geometry and distinguishes between active and passive myocardial motion [8,15,20]. Additionally, GLS correlates strongly with RV ejection fraction (EF) measured by cMR [21] and shows greater reproducibility, with a lower coefficient of variation and higher intra-class correlation coefficient compared to TAPSE and cMR-measured RV function [22]. Furthermore, RVGLS and free wall longitudinal strain (FWLS) have emerged as predictors of prognosis and clinical outcomes across diverse patient cohorts [16,17,23,24,25].

The use of STE has been validated in CCS and is increasingly being used to assess RV function due to its convenience, sensitivity, and reproducibility [26,27]. While invasive mechanical ventilation (IMV) exerts multiple hemodynamic and non-hemodynamic effects on cardiovascular function [28,29,30], there are limited data assessing its acute impact on RV systolic function by STE, particularly in ARF patients. We applied STE retrospectively to echocardiograms in ARF patients requiring IMV to evaluate potential differences in RV systolic function based on the presence or absence of IMV at the time of STE. Our hypothesis is that RVGLS and FWLS will be lower in ARF patients while on IMV compared to when they are not on IMV, even after adjustment for the severity of illness.

## 2. Materials and Methods

This retrospective study was conducted jointly at Rhode Island Hospital and The Miriam Hospital, with approval obtained on 28 January 2018 from the Lifespan institutional review boards (IRBs) of both institutions (approval reference: 201018 45CFR) of the research proposal titled “Left and Right Ventricular Dysfunction by Speckle-Tracking Echocardiography as a Predictor of Outcomes in Respiratory Failure”. Informed consent was waived [26] and the study was conducted according to the ethical principles outlined by the IRB and the Helsinki Declaration.

**Figure 1:**
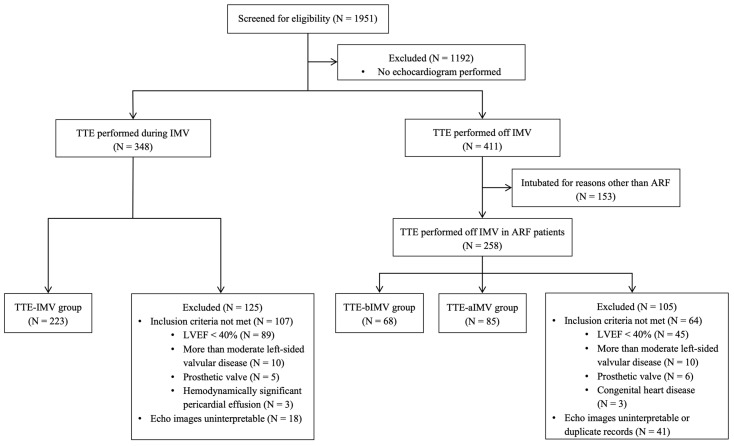
Flowchart of the analytic sample.

All adult patients with a primary diagnosis of ARF requiring IMV for more than 24 h who had transthoracic echocardiography (TTE) during MICU admission between January 2010 and January 2018 were included. Given the retrospective nature of this study, the subjects were divided into three distinct groups based on the timing of TTE performed relative to when they were on IMV: TTE during IMV (TTE-IMV), TTE before IMV (TTE-bIMV), and TTE after IMV (TTE-aIMV). All the subjects had at least one TTE performed during their MICU stay and were categorized according to the first echocardiogram with interpretable images that was performed if none were performed while on IMV [26]. In the patients who had TTE performed while off IMV (TTE-bIMV and TTE-aIMV), the echocardiography closest to the time of IMV was used for the analyses. All the subjects were included in only one subgroup. We removed any duplicates and took whichever echocardiogram had the best images, and if multiple were good, we took the one while on IMV and included that participant in the TTE-IMV group only. The exclusion criteria included (1) pregnancy, (2) intubation for reasons other than ARF (e.g., airway protection), (3) left ventricular ejection fraction (LVEF) less than 40%, (4) more than moderate aortic or mitral valve disease as reported in clinical echocardiography reports, (5) prosthetic heart valves, (6) pericardial effusion with tamponade physiology, (7) congenital heart pathology, or (8) insufficient image quality to perform STE analysis which included inability to visualize the free wall or the inappropriate tracking of more than one wall of RV (Figure 1). The TTE-IMV group had some data reported by this working group in a prior publication [26]. The study is reported according to the Strengthening the Reporting of Observational studies in Epidemiology (STROBE) framework for observational studies [31].

### 2.1. Clinical Variables

The clinical and outcome data, including demographics, social and medical histories, ARF etiology, illness severity score variables (Acute Physiology and Chronic Health Evaluation [APACHE] II), vasopressor and inotrope use, and echocardiography parameters, were collected from each subject’s electronic medical record. The data for APACHE II were obtained within 10 h of echocardiography, and the missing variables were assigned the worst possible value. The recorded data were stored in the Research Electronic Data Capture (RedCap) database.

### 2.2. Traditional 2-D and STE Measurements

Standard TTE and STE measurements were obtained de novo by three trained investigators (S.W., J.S., and Philip Haines), who were blinded to the original echocardiography reports and clinical data. J.S. and Philip Haines measured the RV function parameters in those who had TTE at the time of IMV [26], while S.W and Philip Haines measured these parameters in the subjects not on IMV at the time of TTE (TTE-aIMV and TTE-bIMV). The latter two were blinded to the timing of TTE with respect to IMV. The 2-D and Doppler echocardiography parameters including TAPSE and RVFAC were measured according to the American Society of Echocardiography (ASE) guidelines [11]. LV ejection fraction (LVEF) was obtained from the clinical echocardiography reports.

All the images were analyzed using 2-D cardiac performance analysis version 3.0 (TomTec Imaging Systems, Chicago, IL, USA) in the apical 4-chamber (A 4-C) and subcostal views (SC). RV deep phenotyping was performed according to recommendations from the ASE and the European Association of Cardiovascular Imaging [32,33]. The region of interest (ROI) was obtained by manually tracing the RV endocardium from the lateral to the medial tricuspid annulus along the endocardial border at the end of the diastolic and systolic phases of the cardiac cycle [33]. The RV-focused (A 4-C) was preferred, and the SC was only used if (A 4-C) was suboptimal or not available. The RV strain was reported as an absolute value with lower values correlating to worse strain.

### 2.3. Reliability Analysis for RV Function Parameters

Inter- and intra-class variability was assessed in 10% of the subjects in each group (TTE-IMV and combined TTEaIMV and TTE-bIMV) and was evaluated using predefined cutoffs: poor (<0.5), moderate (0.5–0.75), good (0.75–0.9), and excellent (>0.9) [34]. These were calculated using two-way mixed-effects models.

### 2.4. Statistical Analysis

The continuous variables were summarized as mean and standard deviation for the normally distributed data and as median (interquartile range, IQR) for the non-normally distributed data. The categorical data were presented as frequency and percentages and compared using Pearson’s chi-squared or Fisher’s exact test as appropriate. Logistic and linear regression models were employed to compare the measures of RV function across the TTE-IMV, TTE-bIMV, and TTE-aIMV groups, adjusting for confounders including sex, chronic lung disease, LVEF, and APACHE II score. The TTE-IMV group served as the reference, with differences in means reported as β-coefficients. Statistical significance was set at *p* < 0.05, with analyses conducted using Stata/MP 16.1 (College Station, TX, USA).

## 3. Results

Among the 376 patients admitted to the MICU with ARF requiring IMV and included in the study sample, the TTE-IMV, TTE-bIMV, and TTE-aIMV groups consisted of 223, 68, and 85 subjects, respectively. The median age of the total cohort was 65 years (IQR: 56–74), with 53.2% being male. The clinical characteristics of the three groups were generally similar; however, there were differences in median body mass index, the presence of shock requiring vasopressor medications, and the etiology of ARF (Table 1). Pneumonia was the predominant cause of ARF across all three groups. Additionally, a history of chronic obstructive pulmonary disease or obstructive sleep apnea was more common in the TTE-aIMV group. As expected, the TTE-IMV group had a higher median total APACHE II score compared to the other two groups, and the TTE-bIMV group had a higher median total APACHE II score compared to the TTE-aIMV group.

The TTE-IMV group underwent a TTE with a mean of 1.4 ± 1.9 days after intubation. Conversely, the subjects in the TTE-bIMV and TTE-aIMV groups underwent TTE an average of 3.6 ± 3.9 days before the initiation of IMV and approximately 3.2 ± 2.7 days after extubation, respectively. The median positive end expiratory pressure (PEEP) among the participants in the TTE-IMV group was 8 (IQR 5–10) cmH_2_O. In addition, the mean LVEF of all three groups was normal and was reported to be 59.4 ± 10.6, 62.1 ± 9.8, and 61.1 ± 9.2, for the TTE-IMV, TTE-bIMV, and TTE-aIMV groups, respectively.

The TTE-IMV group had significantly worse RV function compared to the TTE-bIMV and TTE-aIMV groups as assessed by RVGLS, FWLS, and TAPSE (Table 2). A subgroup analysis of the TTE-IMV group showed no significant association between PEEP and any of our echocardiographic measures of RV function, including RVGLS.

### 3.1. Invasive Mechanical Ventilation as a Predictor of RV Function

In the unadjusted multivariable regression analysis assessing the association between IMV and RV function, we found significant differences between the means of the reference group (TTE-IMV) and the other two groups. Specifically, RVGLS was significantly better in the two groups not on IMV at the time of TTE (TTE-bIMV: β = 7.05, 95% CI 5.14 to 8.96, *p* < 0.001; TTE-aIMV: β = 8.04, 95% CI 6.41 to 9.67, *p* < 0.001). Similar significant differences were seen in FWLS (TTE-bIMV: β = 5.88, 95% CI 3.69 to 8.08, *p* < 0.001; TTE-aIMV: β = 7.82, 95% CI 5.82 to 9.81, *p* < 0.001) and TAPSE (TTE-bIMV: β = 2.12, 95% CI 0.49 to 3.75, *p* < 0.01; TTE-aIMV: β = 3.68, 95% CI 2.10 to 5.18, *p* < 0.001) in the TTE-bIMV and TTE-aIMV groups (Table 3).

After adjusting for sex, chronic lung disease, LVEF, and APACHE II score, the differences in the means continued to show significantly better RV function in the groups not on IMV at the time of TTE as measured by RVGLS (TTE-bIMV: β = 7.28, 95% CI 5.07 to 9.48, *p* < 0.001; TTE-aIMV: β = 9.39, 95% CI 6.10 to 10.69, *p* < 0.001) and FWLS (TTE-bIMV: β = 5.83, 95% CI 3.36 to 8.31; TTE-aIMV: β = 7.54, 95% CI 4.83 to 10.24, *p* < 0.001). However, no significant differences were found in TAPSE (TTE-bIMV: β = 0.58, 95% CI −1.25 to 2.41, *p* = 0.53; TTE-aIMV: β = 1.38, 95% CI −0.76 to 3.53, *p* = 0.21) (Table 3).

RVFAC was significantly lower in the TTE-bIMV and TTE-aIMV groups as compared to the TTE-IMV group, and this trend continued after adjusting for covariates. However, FAC remained within the normal range across all three groups (Table 2 and Table 3).

### 3.2. Reliability Analysis

In the cohort that had TTE performed while off IMV, TAPSE showed excellent reliability (ICC = 0.95), while RV-FAC and FWLS showed good reliability (ICC = 0.80 and ICC = 0.84, respectively). GLS had moderate reliability with an ICC of 0.71. In contrast, the inter-class coefficient (ICC*) for RV-GLS and FWLS was moderate (ICC* = 0.66 and ICC* = 0.56, respectively), and RV-FAC and TAPSE was good (ICC* = 0.63 and ICC* = 0.77, respectively). Furthermore, the ICC for the TTE-IMV group revealed excellent reliability for TAPSE (ICC = 0.99), good reliability for GLS (ICC = 0.85), and moderate reliability for FWLS (ICC = 0.66) (Supplementary Table S1) [26].

## 4. Discussion

Our study showed an association between the presence of IMV and lower RVLS (both GLS and FWLS) in critically ill patients requiring IMV during their MICU admission compared to those with ARF who were not on IMV at the time of TTE. It also showed RVGLS and FWLS detected acute RV dysfunction (defined as mean GLS, FWLS, and TAPSE less than 24.5 ± 3.8, 28.5 ± 4.8, and 17 mm, respectively) in the ARF patients admitted to the MICU that was not appreciated by standard TTE parameters like TAPSE and FAC after adjusting for patient factors.

IMV plays a crucial role in modifying cardiopulmonary hemodynamics through various mechanisms. Similarly, numerous studies have proposed that IMV and ARF have direct impacts on RV function [28,29,30,35,36], consistent with our findings. Positive pressure ventilation (PPV) and peak end expiratory pressure (PEEP) increase intrathoracic pressure, leading to reduced venous return and the displacement of the IVS towards the RV [30]. This alteration in cardiac mechanics can affect cardiac output (CO) [29] and patients with pre-existing cardiac dysfunction may experience a more pronounced decrease in CO while on IMV [37]. The impact of PEEP on pulmonary vascular resistance (PVR) and RV afterload can be variable [30], with higher levels of PEEP and hyperinflation generally contributing to significantly elevated PVR. Dessap et al. have reported RV dysfunction secondary to an increase in RV after-load as a result of IMV and PEEP-induced hyperinflation [35]. However, our study did not show any association between PEEP and any measures of RV function. This suggests that factors other than PEEP, such as the mode of mechanical ventilation, tidal volume, peak inspiratory pressure, plateau pressure, and inspiratory flow rate may contribute to changes in RV function during IMV. The right atrium, RV, and vena cava collectively form a low-pressure, high-capacitance unit that is highly sensitive to sudden changes in intrathoracic and transmural pressure [38]. The positive pressure from IMV can exacerbate these pressure changes and exert hemodynamic stress on the right heart system causing the RV to adapt to these changes by dilatation due to lack of contractile reserve [38]. This adaption leads to uncoupling of the RV and the pulmonary circulation [39], which may manifest as impairment in RV function on imaging assessment.

ARF, stemming from diverse pulmonary and extrapulmonary causes, can also adversely impact RV function [40]. The basic underlying mechanism is decreased oxygen delivery to the peripheral tissues leading to tissue injury and death. Severe ARF due to acute respiratory distress syndrome (ARDS) leads to endothelial dysfunction, increased pulmonary vascular permeability, pulmonary vasoconstriction, and vascular remodeling [41]. This can lead to elevated PVR, pre-capillary pulmonary hypertension, and increased RV afterload. In addition to hypoxia and hypercapnia-induced vascular remodeling, pulmonary micro-thrombi and pro-inflammatory cytokine release in response to severe illness have also been shown to contribute to pulmonary vascular remodeling causing RV dysfunction [36].

Although TAPSE was initially significantly lower in the TTE-IMV group in unadjusted models, this difference between the groups became non-significant after adjusting for patient-related covariates, suggesting variables outside of the presence of IMV contribute to TAPSE as well. Additionally, despite a non-significant decrease in TAPSE with IMV, the mean TAPSE remained above the cutoff defined for RV dysfunction (TAPSE < 17 mm) across all groups, so clinicians may overlook the decrease in TAPSE and underestimate the degree of impairment in RV function while on IMV [11]. However, the TTE-IMV group exhibited lower GLS and FWLS compared to the mean normal values defined as 24.5 ± 3.8 and 28.5 ± 4.8, respectively [42], suggesting STE measures may be more sensitive than TAPSE in detecting the presence of RV dysfunction in ARF patients requiring IMV. Although this retrospective study lacks a gold standard comparison to determine sensitivity directly, the consistent identification of RV strain parameters lower than those defined as normal cutoffs in ARF patients, as compared to TAPSE, provides valuable insights into the potential of STE for detecting acute changes in RV function in critically ill patients, particularly those receiving IMV.

Mean FAC, another traditional parameter, was within the normal range across all groups. This finding is consistent with previous studies demonstrating a poor correlation of FAC with RVEF and lower sensitivity and specificity in identifying impaired RV function [43,44].

Among the groups, there were varied etiologies of ARF, chronic comorbid respiratory conditions, and, as a result, expected differences in illness severity. While this potentially confounds the relationship between IMV and RV strain, the differences persisted after multivariable adjustment, including for chronic lung conditions and APACHE. Despite the limitations in ascertaining the etiology of underlying RV function changes, our study underscores the superior ability of RVGLS and FWLS to detect acute changes in RV function compared to TAPSE. This is consistent with prior studies revealing the superiority of GLS and FWLS to detect changes in RV function and also having a better prognostic ability than TAPSE in multiple different patient populations [16,17,23,24,25]. Hence, STE can prove to be a valuable tool in assessing RV function beyond the measurement limitations of TAPSE and the logistical constraints of cMR in ARF patients requiring IMV.

### Study Limitations

There are several limitations to our study. First, this is a single-center retrospective study, limiting its generalizability. Second, the retrospective design of this study imposes limitations on establishing a clear temporal sequence and causality between RV dysfunction and other factors such as illness severity and presence of IMV. Effect estimates were generated after adjusting for known confounders; however, given the retrospective design, the effect estimates may be influenced by confounding from unknown factors which could not be adjusted for. Therefore, while the study provides valuable insights, it is essential to interpret the results with caution. Third, there was no homogenous single time point for measuring illness severity score in all the three groups with respect to IMV, and these scores were also not repeated at any time point during their MICU stay or hospitalization to determine the highest severity of illness for these patients while in the MICU, although this “noise” would tend to bias results to the null. In addition, not all clinical data for illness severity by APACHE II was recorded in the chart for review, and in those cases the subject was given the lowest illness severity score for that measure, likely underestimating the illness severity across all the groups—the most conservative approach which again would bias to the null. As our study was retrospective, only patients with available echocardiograms during their MICU stay were included, which may have introduced selection bias. Moreover, several different software options for STE are available and we used the vendor-independent TomTec software package (version 3.0, Chicago, IL, USA) for all the cases. Last, we want to acknowledge that despite recognizing the importance of conducting a prospective evaluation to validate our initial findings since the end of data collection, unforeseen circumstances, including the spillover effects of the COVID-19 pandemic, have posed significant challenges. These challenges include limitations in staffing, resources, and access to patients for clinical studies that continue to affect research efforts. Our belief is these findings will continue to add to the mounting evidence that cardiac strain measurements in critically ill patients are feasible, sensitive, and provide a novel understanding of cardiopulmonary pathophysiology, in hopes that important further studies are appropriately supported.

## 5. Conclusions

Our study represents a novel contribution by demonstrating the association between the presence of IMV and lower RVGLS and FWLS, consistent with RV dysfunction, in critically ill patients with ARF. Furthermore, we highlighted that RVLS is likely more sensitive at detecting subtle but potentially clinically significant changes in RV function during IMV and ARF, which were undetected by traditional parameters and standard diagnostic echocardiographic cut-offs in this study. However, future prospective studies incorporating TTE assessments before, after, and during IMV are warranted to clarify the primary driver for the associated worse RVGLS and FWLS in patients with ARF on IMV, and also to evaluate the prognostic value of STE-detected RV dysfunction in this population.

## Figures and Tables

**Table 1:** Baseline characteristics of total cohort who had transthoracic echocardiogram (TTE) performed while on invasive mechanical ventilation (TTE-IMV), before (TTE-bIMV), and after being on invasive mechanical ventilation (TTE-aIMV).

N = 376	Total Cohort (N = 376)	TTE-IMV (N = 223)	TTE-bIMV (N = 68)	TTE-aIMV (N = 85)
Age	(n = 375)	(n = 223)	(n = 67)	(n = 85)
Median (Q1–Q3)	65 (56–74)	65 (55–74)	71 (57–76)	63 (57–74)
Sex				
Male	200 (53.2)	121 (54.3)	39 (57.4)	40 (47.1)
Documented smoking history	(n = 354)	(n = 201)	(n = 68)	(n = 85)
Yes	230 (64.9)	126 (62.7)	51 (75.0)	53 (62.4)
BMI (kg/m^2^)				
Median (Q1–Q3)	28.9 (24.3–35.6)	29.7 (25.2–37.1)	27.8 (22.9–35.3)	28.2 (22.8–33.1)
Chronic lung disease *				
Chronic obstructive lung disease	138 (36.7)	71 (31.8)	26 (38.2)	41 (48.2)
Asthma	28 (7.5)	13 (5.8)	6 (8.8)	9 (10.6)
Interstitial lung disease	10 (2.7)	5 (2.2)	1 (1.5)	4 (4.7)
Cystic fibrosis	1 (0.27)	1 (0.45)	0 (--)	0 (--)
Pulmonary hypertension	18 (4.8)	12 (5.4)	2 (2.9)	4 (4.7)
Obstructive sleep apnea	51 (13.6)	22 (9.9)	12 (17.7)	17 (20.0)
History of primary lung cancer	(n = 375)	(n = 222)	(n = 68)	(n = 85)
Yes	19 (5.1)	9 (4.1)	4 (5.9)	6 (7.1)
Chronic cardiac disease *^	280 (74.5)	159 (71.3)	58 (85.3)	63 (74.1)
Chronic renal disease *^#^	47 (12.5)	32 (14.4)	7 (10.3)	8 (9.4)
Shock requiring pressor support	249 (66.2)	162 (72.7)	44 (64.7)	43 (50.6)
Number of pressors used	(n = 249)	(n = 162)	(n = 44)	(n = 43)
1	125 (50.2)	73 (45.1)	21 (47.7)	31 (72.1)
2	84 (33.7)	58 (35.8)	16 (36.4)	10 (23.3)
3	40 (16.1)	31 (19.1)	7 (15.9)	2 (4.7)
Inotropes	(n = 375)	(n = 223)	(n = 68)	(n = 84)
Yes	18 (4.8)	11 (4.9)	5 (7.4)	2 (2.4)
Total APACHE II score	(n = 364)	(n = 223)	(n = 64)	(n = 77)
Median (Q1–Q3)	18 (12–24)	22 (17–27)	13.5 (10–19)	11 (6–15)
Etiology of acute respiratory failure	(n = 375)	(n = 222)	(n = 68)	(n = 85)
Pneumonia	120 (32.0)	82 (36.9)	19 (27.9)	19 (22.4)
Pulmonary edema	64 (17.1)	34 (15.3)	17 (25.0)	13 (15.3)
Cardiac arrest	60 (16.0)	46 (20.7)	2 (2.9)	12 (14.1)
Aspiration	54 (14.4)	29 (13.1)	15 (22.1)	10 (11.8)
Acute exacerbation COPD	24 (6.4)	6 (2.7)	3 (4.4)	15 (17.7)
Encephalopathy	15 (4.0)	5 (2.3)	4 (5.9)	6 (7.1)
Other	38 (10.1)	20 (9.0)	8 (11.8)	10 (11.8)
ARDS diagnosed	(n = 369)	(n = 223)	(n = 62)	(n = 84)
Yes	37 (10.0)	21 (9.4)	10 (16.1)	6 (7.1)

Categorical data are N (%). * checked all that applied for a given patient; does not sum to 100%. ^ includes ischemic and non-ischemic cardiomyopathy, valvular heart disease, chronic arrhythmias, or chronic pericarditis. ^#^ includes > stage III or chronic hemodialysis.

**Table 2:** Echocardiography measurements for subjects in TTE-IMV, TTE-bIMV, and TTE-aIMV groups. Absolute strain values are reported with lower values correlating to worse strain.

N = 376	Total Cohort (N = 376)	TTE-IMV (N = 223)	TTE-bIMV (N = 68)	TTE-aIMV (N = 85)	*p*-Value ^1^
RV GLS					
Median (Q1–Q3)	19.0 (13.5–24.7)	15.9 (10.6–20.8)	22.3 (19.3–22.3)	23.8 (19.8–27.0)	<0.001
TAPSE (mm)	(n = 295)	(n = 163)	(n = 57)	(n = 75)	
Median (Q1–Q3)	20 (16.1–23.7)	18.1 (15.4–21.6)	21.6 (17.3–24.1)	22.9 (19.4–25.2)	<0.001
RV FWLS					
Median (Q1–Q3)	23.2 (16.6–28.7)	19.7 (14.0–25.8)	25.5 (21.4–32.0)	27.7 (22.9–31.8)	<0.001
RV FAC	(n = 313)	(n = 160)	(n = 68)	(n = 85)	
Median (Q1–Q3)	46.7 (37.9–55.1)	51.3 (42.0–59.9)	40.8 (34.9–49.6)	44.1 (36.4–51.9)	<0.001

^1^ Wilcoxon rank-sum.

**Table 3:** Unadjusted and adjusted models of RV function parameters.

TTE Parameters	Unadjusted β (95% CI)	*p*-Value	Adjusted β * (95% CI)	*p*-Value
RV GLS				
TTE-IMV	Reference	Reference	Reference	Reference
TTE-bIMV	7.05 (5.14, 8.96)	<0.001	7.28 (5.07, 9.48)	<0.001
TTE-aIMV	8.04 (6.41, 9.67)	<0.001	9.39 (6.10, 10.69)	<0.001
TAPSE				
TTE-IMV	Reference	Reference	Reference	Reference
TTE-bIMV	2.12 (0.49, 3.75)	0.01	0.58 (−1.25, 2.41)	0.53
TTE-aIMV	3.68 (2.10, 5.18)	<0.001	1.38 (−0.76, 3.53)	0.21
RV FWLS				
TTE-IMV	Reference	Reference	Reference	Reference
TTE-bIMV	5.88 (3.69, 8.08)	<0.001	5.83 (3.36, 8.31)	<0.001
TTE-aIMV	7.82 (5.82, 9.81)	<0.001	7.54 (4.83, 10.24)	<0.001
RV FAC				
TTE-IMV	Reference	Reference	Reference	Reference
TTE-bIMV	−9.76 (−13.23, −6.28)	<0.001	−11.32 (−15.29, −7.35)	<0.001
TTE-aIMV	−6.91 (−9.99, −3.82)	<0.001	−9.14 (−13.48, −4.81)	<0.001

* adjusted for sex, chronic lung disease, left ventricular ejection fraction, and APACHE II score.

## Data Availability

The data used in the analysis are available upon request to the corresponding author.

## Supplementary Materials

The following supporting information can be downloaded at: https://www.mdpi.com/article/10.3390/jcdd11080246/s1; Table S1: Reliability assessment of speckle tracking and traditional echocardiography parameters.

## References

[1] Vieillard-Baron A., Naeije R., Haddad F., Bogaard H.J., Bull T.M., Fletcher N., Lahm T., Magder S., Orde S., Schmidt G. (2018). Diagnostic workup, etiologies and management of acute right ventricle failure: A state-of-the-art paper. Intensive Care Med..

[2] Sato R., Cheungpasitporn W., Schleicher M., Collier P., Vallabhajosyula S., Duggal A. (2021). The impact of right ventricular injury on the mortality in patients with acute respiratory distress syndrome: A systematic review and meta-analysis. Crit. Care.

[3] Vieillard-Baron A., Schmitt J.M., Augarde R., Fellahi J.L., Prin S., Page B., Beauchet A., Jardin F. (2001). Acute cor pulmonale in acute respiratory distress syndrome submitted to protective ventilation: Incidence, clinical implications, and prognosis. Crit. Care Med..

[4] Boissier F., Katsahian S., Razazi K., Thille A.W., Roche-Campo F., Leon R., Vivier E., Brochard L., Vieillard-Baron A., Brun-Buisson C. (2013). Prevalence and prognosis of cor pulmonale during protective ventilation for acute respiratory distress syndrome. Intensive Care Med..

[5] Lheritier G., Legras A., Caille A., Lherm T., Mathonnet A., Frat J.P., Courte A., Martin-Lefèvre L., Gouëllo J.P., Amiel J.B. (2013). Prevalence and prognostic value of acute cor pulmonale and patent foramen ovale in ventilated patients with early acute respiratory distress syndrome: A multicenter study. Intensive Care Med..

[6] Wheeler A.P., Bernard G.R., Thompson B.T., Schoenfeld D., Wiedemann H.P., de Boisblanc B., Connors A.F., Hite R.D., Harabin A.L., National Heart, Lung, and Blood Institute Acute Respiratory Distress Syndrome (ARDS) Clinical Trials Network (2006). Pulmonary-artery versus central venous catheter to guide treatment of acute lung injury. N. Engl. J. Med..

[7] Richard C., Warszawski J., Anguel N., Deye N., Combes A., Barnoud D., Boulain T., Lefort Y., Fartoukh M., Baud F. (2003). Early use of the pulmonary artery catheter and outcomes in patients with shock and acute respiratory distress syndrome: A randomized controlled trial. JAMA.

[8] Pastorini, Anastasio F., Feola M. (2023). What Strain Analysis Adds to Diagnosis and Prognosis in Heart Failure Patients. J. Clin. Med..

[9] Miller D., Farah M.G., Liner A., Fox K., Schluchter M., Hoit B.D. (2004). The relation between quantitative right ventricular ejection fraction and indices of tricuspid annular motion and myocardial performance. J. Am. Soc. Echocardiogr..

[10] Rudski L.G., Lai W.W., Afilalo J., Hua L., Handschumacher M.D., Chandrasekaran K., Solomon S.D., Louie E.K., Schiller N.B. (2010). Guidelines for the echocardiographic assessment of the right heart in adults: A report from the American Society of Echocardiography endorsed by the European Association of Echocardiography, a registered branch of the European Society of Cardiology, and the Canadian Society of Echocardiography. J. Am. Soc. Echocardiogr..

[11] Lang R.M., Badano L.P., Mor-Avi V., Afilalo J., Armstrong A., Ernande L., Flachskampf F.A., Foster E., Goldstein S.A., Kuznetsova T. (2015). Recommendations for cardiac chamber quantification by echocardiography in adults: An update from the American Society of Echocardiography and the European Association of Cardiovascular Imaging. Eur. Heart J. Cardiovasc. Imaging..

[12] Focardi M., Cameli M., Carbone S.F., Massoni A., De Vito R., Lisi M., Mondillo S. (2015). Traditional and innovative echocardiographic parameters for the analysis of right ventricular performance in comparison with cardiac magnetic resonance. Eur. Heart J. Cardiovasc. Imaging..

[13] Lemarie J., Huttin O., Girerd N., Mandry D., Juillière Y., Moulin F., Lemoine S., Beaumont M., Marie P.Y., Selton-Suty C. (2015). Usefulness of Speckle-Tracking Imaging for Right Ventricular Assessment after Acute Myocardial Infarction: A Magnetic Resonance Imaging/Echocardiographic Comparison within the Relation between Aldosterone and Cardiac Remodeling after Myocardial Infarction Study. J. Am. Soc. Echocardiogr..

[14] Wong A., Galarza L., Forni L., De Backer D., Slama M., Cholley B., Mayo P., McLean A., Vieillard-Baron A., Lichtenstein D. (2020). Recommendations for core critical care ultrasound competencies as a part of specialist training in multidisciplinary intensive care: A framework proposed by the European Society of Intensive Care Medicine (ESICM). Crit. Care.

[15] Hahn R.T., Lerakis S., Delgado V., Addetia K., Burkhoff D., Muraru D., Pinney S., Friedberg M.K. (2023). Multimodality Imaging of Right Heart Function: JACC Scientific Statement. J. Am. Coll. Cardiol..

[16] Carluccio E., Biagioli P., Alunni G., Murrone A., Zuchi C., Coiro S., Riccini C., Mengoni A., D’Antonio A., Ambrosio G. (2018). Prognostic Value of Right Ventricular Dysfunction in Heart Failure with Reduced Ejection Fraction: Superiority of Longitudinal Strain Over Tricuspid Annular Plane Systolic Excursion. Circ. Cardiovasc. Imaging.

[17] Ternacle J., Berry M., Cognet T., Kloeckner M., Damy T., Monin J.L., Couetil J.P., Dubois-Rande J.L., Gueret P., Lim P. (2013). Prognostic value of right ventricular two-dimensional global strain in patients referred for cardiac surgery. J. Am. Soc. Echocardiogr..

[18] Vieillard-Baron A., Prigent A., Repessé X., Goudelin M., Prat G., Evrard B., Charron C., Vignon P., Geri G. (2020). Right ventricular failure in septic shock: Characterization, incidence and impact on fluid responsiveness. Crit. Care.

[19] Lee J.H., Park J.H. (2018). Strain Analysis of the Right Ventricle Using Two-dimensional Echocardiography. J. Cardiovasc. Imaging.

[20] Potter E., Marwick T.H. (2018). Assessment of Left Ventricular Function by Echocardiography: The Case for Routinely Adding Global Longitudinal Strain to Ejection Fraction. JACC Cardiovasc. Imaging.

[21] Vizzardi E., Bonadei I., Sciatti E., Pezzali N., Farina D., D’Aloia A., Metra M. (2015). Quantitative analysis of right ventricular (RV) function with echocardiography in chronic heart failure with no or mild RV dysfunction: Comparison with cardiac magnetic resonance imaging. J. Ultrasound Med..

[22] Houard L., Militaru S., Tanaka K., Pasquet A., Vancraeynest D., Vanoverschelde J.L., Pouleur A.C., Gerber B.L. (2021). Test-retest reliability of left and right ventricular systolic function by new and conventional echocardiographic and cardiac magnetic resonance parameters. Eur. Heart J. Cardiovasc. Imaging..

[23] Ingvarsson A., Werther Evaldsson A., Waktare J., Nilsson J., Smith G.J., Stagmo M., Roijer A., Rådegran G., Meurling C.J. (2018). Normal Reference Ranges for Transthoracic Echocardiography Following Heart Transplantation. J. Am. Soc. Echocardiogr..

[24] Orde S.R., Pulido J.N., Masaki M., Gillespie S., Spoon J.N., Kane G.C., Oh J.K. (2014). Outcome prediction in sepsis: Speckle tracking echocardiography based assessment of myocardial function. Crit. Care.

[25] Hestenes S.M., Halvorsen P.S., Skulstad H., Remme E.W., Espinoza A., Hyler S., Bugge J.F., Fosse E., Nielsen E.W., Edvardsen T. (2014). Advantages of strain echocardiography in assessment of myocardial function in severe sepsis: An experimental study. Crit. Care Med..

[26] Simmons J., Haines P., Extein J., Bashir Z., Aliotta J., Ventetuolo C.E. (2022). Systolic Strain by Speckle-Tracking Echocardiography Is a Feasible and Sensitive Measure of Right Ventricular Dysfunction in Acute Respiratory Failure Patients on Mechanical Ventilation. Crit. Care Explor..

[27] Vieillard-Baron A., Millington S.J., Sanfilippo F., Chew M., Diaz-Gomez J., McLean A., Pinsky M.R., Pulido J., Mayo P., Fletcher N. (2019). A decade of progress in critical care echocardiography: A narrative review. Intensive Care Med..

[28] Vieillard-Baron A., Loubieres Y., Schmitt J.M., Page B., Dubourg O., Jardin F. (1999). Cyclic changes in right ventricular output impedance during mechanical ventilation. J. Appl. Physiol. (1985).

[29] Mahmood S.S., Pinsky M.R. (2018). Heart-lung interactions during mechanical ventilation: The basics. Ann. Transl. Med..

[30] Corp A., Thomas C., Adlam M. (2021). The cardiovascular effects of positive pressure ventilation. BJA Educ..

[31] Cuschieri S. (2019). The STROBE guidelines. Saudi J. Anaesth..

[32] Mor-Avi V., Lang. R.M., Badano L.P., Belohlavek M., Cardim N.M., Derumeaux G., Galderisi M., Marwick T., Nagueh S.F., Sengupta P.P. (2011). Current and evolving echocardiographic techniques for the quantitative evaluation of cardiac mechanics: ASE/EAE consensus statement on methodology and indications endorsed by the Japanese Society of Echocardiography. J. Am. Soc. Echocardiogr..

[33] Badano L.P., Kolias T.J., Muraru D., Abraham T.P., Aurigemma G., Edvardsen T., D’Hooge J., Donal E., Fraser A.G., Marwick T. (2018). Standardization of left atrial, right ventricular, and right atrial deformation imaging using two-dimensional speckle tracking echocardiography: A consensus document of the EACVI/ASE/Industry Task Force to standardize deformation imaging. Eur. Heart J. Cardiovasc. Imaging..

[34] Vos M.E., Cox E.G.M., Schagen M.R., Hiemstra B., Wong A., Koeze J., van der Horst I.C.C., Wiersema R., SICS Study Group (2022). Right ventricular strain measurements in critically ill patients: An observational SICS sub-study. Ann. Intensive Care.

[35] Mekontso Dessap A., Boissier F., Charron C., Bégot E., Repessé X., Legras A., Brun-Buisson C., Vignon P., Vieillard-Baron A. (2016). Acute cor pulmonale during protective ventilation for acute respiratory distress syndrome: Prevalence, predictors, and clinical impact. Intensive Care Med..

[36] Grignola J.C., Domingo E. (2017). Acute Right Ventricular Dysfunction in Intensive Care Unit. Biomed. Res. Int..

[37] Bindslev L., Hedenstierna G., Santesson J., Gottlieb I., Carvallhas A. (1981). Ventilation-perfusion distribution during inhalation anaesthesia. Effects of spontaneous breathing, mechanical ventilation and positive end-expiratory pressure. Acta Anaesthesiol. Scand..

[38] Spruijt O.A., de Man F.S., Groepenhoff H., Oosterveer F., Westerhof N., Vonk-Noordegraaf A., Bogaard H.J. (2015). The effects of exercise on right ventricular contractility and right ventricular-arterial coupling in pulmonary hypertension. Am. J. Respir. Crit. Care Med..

[39] Petit M., Jullien E., Vieillard-Baron A. (2021). Right Ventricular Function in Acute Respiratory Distress Syndrome: Impact on Outcome, Respiratory Strategy and Use of Veno-Venous Extracorporeal Membrane Oxygenation. Front. Physiol..

[40] Mirabile V.S., Shebl E., Sankari A., Sankari A., Burns B. Respiratory Failure in Adults. In: StatPearls. Treasure Island (FL): StatPearls Publishing; 2024 Jan-. https://www.ncbi.nlm.nih.gov/books/NBK526127/.

[41] Price L.C., McAuley D.F., Marino P.S., Finney S.J., Griffiths M.J., Wort S.J. (2012). Pathophysiology of pulmonary hypertension in acute lung injury. Am. J. Physiol. Lung Cell Mol. Physiol..

[42] Morris D.A., Krisper M., Nakatani S., Köhncke C., Otsuji Y., Belyavskiy E., Radha Krishnan A.K., Kropf M., Osmanoglou E., Boldt L.H. (2017). Normal range and usefulness of right ventricular systolic strain to detect subtle right ventricular systolic abnormalities in patients with heart failure: A multicentre study. Eur. Heart J. Cardiovasc. Imaging.

[43] Barthelemy R., Roy X., Javanainen T., Mebazaa A., Chousterman B.G. (2019). Comparison of echocardiographic indices of right ventricular systolic function and ejection fraction obtained with continuous thermodilution in critically ill patients. Crit. Care.

[44] Prihadi E.A., van der Bijl P., Dietz M., Abou R., Vollema E.M., Marsan N.A., Delgado V., Bax J.J. (2019). Prognostic Implications of Right Ventricular Free Wall Longitudinal Strain in Patients With Significant Functional Tricuspid Regurgitation. Circ. Cardiovasc. Imaging.

